# Chemical Constituents of the Leaves of Butterbur (*Petasites japonicus*) and Their Anti-Inflammatory Effects

**DOI:** 10.3390/biom9120806

**Published:** 2019-11-29

**Authors:** Jin Su Lee, Miran Jeong, Sangsu Park, Seung Mok Ryu, Jun Lee, Ziteng Song, Yuanqiang Guo, Jung-Hye Choi, Dongho Lee, Dae Sik Jang

**Affiliations:** 1 Department of Life and Nanopharmaceutical Sciences, Graduate School, Kyung Hee University, Seoul 02447, Korea; lee2649318@naver.com (J.S.L.); jchoi@khu.ac.kr (J.-H.C.); 2 College of Pharmacy, Kyung Hee University, Seoul 02447, Korea; jeongmiran@hanmail.net; 3 Department of Fundamental Pharmaceutical Sciences, Graduate School, Kyung Hee University, Seoul 02447, Korea; x-zara@nate.com; 4 Herbal Medicine Resources Research Center, Korea Institute of Oriental Medicine, Jeollanam-do 58245, Korea; smryu@kiom.re.kr (S.M.R.); junlee@kiom.re.kr (J.L.); 5 State Key Laboratory of Medicinal Chemical Biology, College of Pharmacy, Tianjin Key Laboratory of Molecular Drug Research, and Drug Discovery Center for Infectious Disease, Nankai University, Tianjin 300350, China; kdszt152@163.com (Z.S.); victgyq@nankai.edu.cn (Y.G.); 6 Department of Biosystems and Biotechnology, College of Life Sciences and Biotechnology, Korea University, Seoul 02841, Korea

**Keywords:** *Petasites japonicus*, Asteraceae, lignan, anti-inflammation, NO, PGE_2_, iNOS, COX-2, molecular docking

## Abstract

Two new aryltetralin lactone lignans, petasitesins A and B were isolated from the hot water extract of the leaves of butterbur (*Petasites japonicus*) along with six known compounds. The chemical structures of lignans **1** and **2** were elucidated on the basis of 1D and 2D nuclear magnetic resonance (NMR) spectroscopic data, electronic circular dichroism (ECD) and vibrational circular dichroism (VCD) spectra. Petasitesin A and cimicifugic acid D showed significant inhibitory effects on the production of both prostaglandin E2 (PGE_2_) and NO in RAW264.7 macrophages. The expressions of inducible nitric oxide synthase (iNOS) and cyclooxygenase-2 (COX-2) were inhibited by compound **1** in RAW264.7 cells. Furthermore, compounds **1** and **3** exhibited strong affinities with both iNOS and COX-2 enzymes in molecular docking studies.

## 1. Introduction

**Figure 1:**
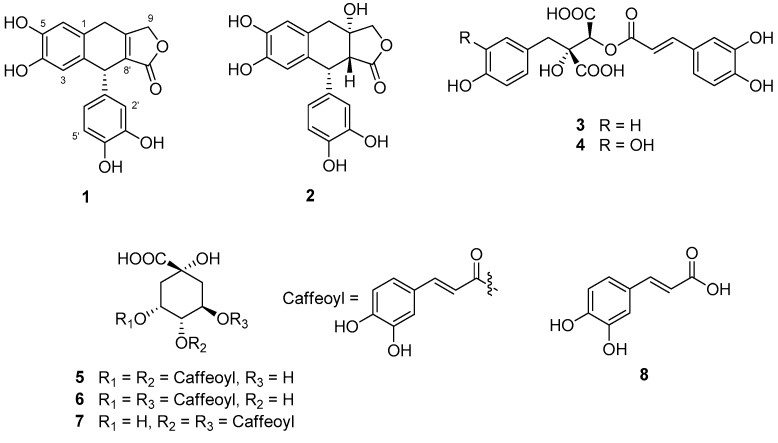
Chemical structures of the isolates **1**–**8**.

*Petasites japonicus* Maxim (Asteraceae), known as butterbur, Japanese butterbur, and giant butterbur, is used as a botanical dietary supplement in the USA. The aerial parts of *P. japonicus* have been used in traditional Japanese folk medicine as an antipyretic, antitussive, or wound healing agent [1]. The constituents of *P. japonicus* have been reported and include flavonoids [2], sesquiterpenes [3,4,5], triterpenes [6], and various types of phenolic compounds [7]. Moreover, the leaves or stalks of *P. japonicus* are commonly consumed as vegetables in Korea and Japan. In the course of searching for active compounds from higher plants [8,9], the leaves of *P. japonicus* were selected for a detailed study since a hot water extract of the leaves of *P. japonicus* have shown inhibitory activity against nitric oxide (NO) production in RAW 264.7 cells [half maximal inhibitory concentration (IC_50_) value: 19 ± 4.9 µg/mL]. Phytochemical study on the hot water extract resulted in the isolation of two new aryltetralin lactone lignans (**1** and **2**) along with six previously known compounds (Figure 1). The chemical structures of the new lignans **1** and **2** were determined by interpretation of 1D and 2D nuclear magnetic resonance (NMR) spectroscopic data, and by electronic circular dichroism (ECD) and vibrational circular dichroism (VCD) studies. 

All the isolates from the leaves of *P. japonicus* were evaluated for their inhibitory effects on lipopolysaccharide (LPS)-induced production of pro-inflammatory mediators NO and prostaglandin E_2_ (PGE_2_) in the LPS-stimulated RAW 264.7 macrophages. We describe isolation of the secondary metabolites from the leaves of *P. japonicus*, structure elucidation of the two new lignans (**1** and **2**), and anti-inflammatory effects of the isolates as well as the possible mechanism.

## 2. Materials and Methods 

### 2.1. General Experimental Procedures

General experimental procedures are described in the Supplementary Materials.

### 2.2. Plant Material

The leaves of *Petasites japonicus* (Asteraceae) were obtained from Nature Bio Co. (Seoul, Republic of Korea), in October 2016. The plant material was identified by one of the authors (D.S.J.) and the plant specimen (PEJA-2016) has been deposited in the Laboratory of Natural Product Medicine, College of Pharmacy, Kyung Hee University.

### 2.3. Isolation of Compounds

The dried leaves (500 g) were extracted once with 10 L of boiled water for 4 h and the solvent was evaporated with freeze drying. The extract (100.0 g) was separated over Diaion HP-20 (Mitsubishi, Tokyo, Japan) column eluted with an H_2_O-acetone gradient (from 1:0 to 0:1 *v/v*, gradient) to give 15 fractions (K1–K15). A part of fraction K3 was fractionated with medium pressure liquid chromatography (MPLC) using Redi Sep (Teledyne Isco, Lincoln, NE, USA)-C18 cartridge (13 g, acetonitrile–H_2_O, 0:1 to 3:7 *v/v*, gradient) and purified by high performance liquid chromatography (HPLC) using YMC Pack ODS-A column (Phenomenex, Torrance, CA, USA), yielding compound **8** (7.7 mg). Fraction K6 was separated over Sephadex LH-20 (Amersham Pharmacia Biotech, Buckinghamshire, United Kingdom) column with an acetone–H_2_O mixture (6:4 *v/v*) as solvent to give three fractions (K6-1–K6-3). Fraction K6-2 was fractionated further using Sephadex LH-20 with an acetone–H_2_O mixture (2:8 *v/v*) to yield six subfractions (K6-2-1–K6-2-6). Compound **4** (0.5 g) and **3** (27.1 mg) were obtained from fraction K6-2-4 using LiChroprep RP-18 (Merck, Kenilworth, NJ, USA) CC. Fraction K8 was loaded to Sephadex LH-20 as stationary phase eluting with EtOH–H_2_O mixture (1:1 *v/v*) to afford 12 pooled fractions (K8-1–K8-12). Compound **5** (40.3 mg), **6** (4.2 mg), and **7** (4.7 mg) were purified from fraction K8-6 by HPLC with a Luna 10 μm C18(2) 100 Å column. Subfraction K8-9 was purified using Luna 10 μm C18 (**2**) 100 Å column to obtain compound **2** (61.6 mg). Fraction K9 was fractionated further using Sephadex LH-20 column eluted with the EtOH–H_2_O mixture (1:1 *v/v*) to generate ten fractions (K9-1–K9-10). Fraction K9-6 was purified by HPLC using YMC Pack ODS-A column, yielding compound **1** (4.2 mg).

#### 2.3.1. Petasitesin A (**1**)

Dark brownish powder; [α]_D_^23^: −11.5° (*c* 0.1, MeOH); ultraviolet (UV) (MeOH) λ_max_ (log *ε*) 204 nm (3.85), 262 nm (3.57); CD (CH_3_CN) λ_max_ 214 (−10.4), 239 (2.1), 254 (−5.3); infrared (IR) (ATR) ν_max_ 3333, 2915, 2847, 1718, 1524, 1240 cm^−1^; High resolution electrospray ionization mass spectrometry (HRESIMS) (HRESIMS) (negative mode) *m*/*z* 325.0714 [M−H]^−^ (calculated for C_18_H_13_O_6_, 325.0712) (Figure S1); ^1^H and ^13^C NMR data (Table 1) (Figures S2 and S3); 2D NMR data (Figures S4–S7).

#### 2.3.2. Petasitesin B (**2**)

Dark brownish powder; [α]_D_^23^: −20.6° (*c* 0.1, MeOH); UV (MeOH) λ_max_ (log *ε*) 206 nm (4.34), 287 nm (3.48); CD (CH_3_CN) λ_max_ 203 (4.0), 210 (−5.4), 222 (−4.4), and 229 (1.9); IR (ATR) ν_max_ 3305, 1768, 1606, 1514 cm^−1^; HRESIMS (negative mode) *m*/*z* 343.0810 [M−H]^−^ (calculated for C_18_H_15_O_7_, 343.0818) (Figure S8); ^1^H and ^13^C NMR data (Table 1) (Figures S9 and S10); 2D NMR data (Figures S11–S14).

### 2.4. Computational Methods

ECD and VCD calculations of compounds **1** and **2** were conducted as described previously [10,11]. In brief, their 3D models were built from Chem3D modeling. Conformational analysis was performed by the MMFF force field as implemented in Spartan’14 software (Wavefunction, Inc., Irvine, CA, USA; 2014). Geometrical optimization of the selected conformers was performed at the B3LYP/6–31 + G (d,p) level by Gaussian 09 software (Revision E.01; Gaussian, Inc., Wallingford, CT, USA; 2009). The theoretical ECD and VCD spectra were calculated at the CAM-B3LYP/SVP level with a CPCM solvent model (acetonitrile) and at the DFT [B3LYP/6–31 + G(d,p)] basis set level by the Gaussian 09 software, respectively.

### 2.5. Measurement of NO Production

The 3-[4¨C-dimethylthiazol-2-yl]-2,5-dipheyl tetrazolium bromide (MTT) and Griess reaction assays were used for cell viability studies and measuring nitrite levels, respectively, as reported previously [12]. 

### 2.6. Measurement of PGE_2_

The RAW 264.7 macrophage cell lines were pretreated with various concentrations of the extract and isolates **1**–**8** for 1 h and then stimulated with or without LPS (1 μg/mL) for 24 h. A selective COX-2 inhibitor, NS-398 (*N*-[2-(cyclohexyloxy)-4-nitrophenyl]methanesulfonamide; Sigma Aldrich, St. Louis, MO, USA) was used as a positive control for blocking PGE_2_ production. PGE_2_ levels in cell culture mediums were measured using the same methods as described in the previous paper [12].

### 2.7. Measurement of iNOS and COX-2 Expression

Quantitative polymerase chain reaction (qPCR) using Thermal Cycler Dice Real Time PCR System (Takara Bio Inc., Shiga, Japan) was used to determine the steady-state mRNA levels of inducible nitric oxide synthase (iNOS) and cyclooxygenase-2 (COX-2) as reported previously [12].

### 2.8. Molecular Docking Studies

The software AutoDock Vina with AutoDock Tools (The Scripps Research Institute, La Jolla, CA, USA: ADT 1.5.6) using the hybrid Lamarckian Genetic Algorithm (LGA) was used for performing molecular docking simulations as reported in the literature [13,14]. In short, the 3D crystal structures (resolution: 2.5 Å) of iNOS (PDB code: 3E6T) and COX-2 (PDB code: 1PXX) were obtained from the RCSB Protein Data Bank. The configurations of compounds **1** and **3** were determined by their nuclear overhauser effect spectroscopy (NOESY) spectra and time-dependent density functional theory (TDDFT) ECD calculations. Chem3D Pro 14.0 software (CambridgeSoft, Waltham, MA, USA) was used for construction of the standard 3D structures (PDB format) of compounds **1** and **3**. 

## 3. Results

### 3.1. Structure Elucidation of Compounds **1** and **2**

Compound **1** was obtained as a dark brownish powder, and its molecular formula was identified as C_18_H_14_O_6_ by HRESIMS (*m/z* 325.0714 [M−H]^−^; calculated for C_18_H_13_O_6_, 325.0712). It exhibited UV maxima at 262 nm and IR maxima at 3333, 1718, and 1524 cm^-1^, suggesting the presence of a hydroxyl, ester group, and aromatic ring. The ^13^C NMR spectral data of compound **1** (Table 1) exhibited 18 carbon signals including a carbonyl carbon (*δ*_C_ 173.9), 12 aromatic carbons (from *δ*_C_ 115.7 to 145.9), an oxygenated methylene carbon (*δ*_C_ 72.4), a methine carbon (*δ*_C_ 42.3), and a methylene carbon (*δ*_C_ 29.0). 

**Figure 2:**
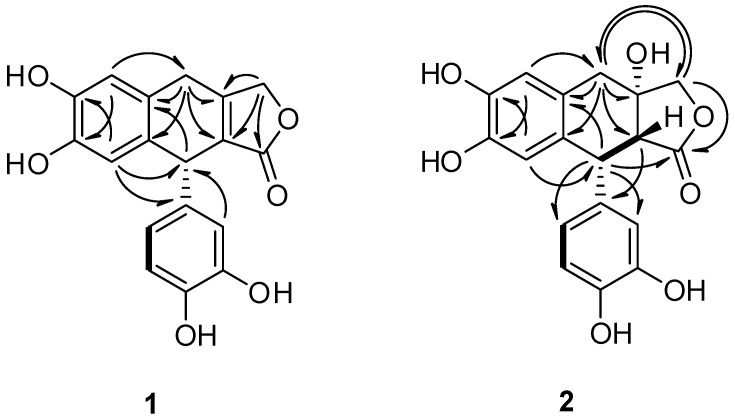
Selected correlations of compounds **1** and **2**: correlation spectroscopy (COSY, ▬) and heteronuclear multiple bond correlation spectroscopy (HMBC, →) (in acetone-*d*_6_ and methanol-*d*_4_).

The remaining two quaternary carbons (*δ*_C_ 160.9 and 128.2) were derived from a double bond. The ^1^H NMR spectrum revealed one 1,2,4,5-tetrasubstituted aromatic ring [*δ*_H_ 6.72 (s, H-6) and 6.60 (s, H-3)], and the one 1,3,4-trisubstituted aromatic ring [*δ*_H_ 6.63 (d, *J* = 8.0, H-5′), 6.59 (d, *J* = 2.0, H-2′), and 6.45 (dd, *J* = 8.0 and 2.0, H-6′)], an oxygenated methylene [*δ*_H_ 4.97 (d, *J* = 17.0) and 4.89 (d, *J* = 17.5), H-9], a methine [*δ*_H_ 4.53 (s, H-7′)], and a methylene [*δ*_H_ 3.86 (d, *J* = 23.0) and 3.62 (overlapped), H-7]. The heteronuclear multiple bond correlation spectroscopy (HMBC) correlations of **1** (Figure 2) suggest aryltetralin lactone type lignan with a double bond at C-8 and C-8′.

**Figure 3:**
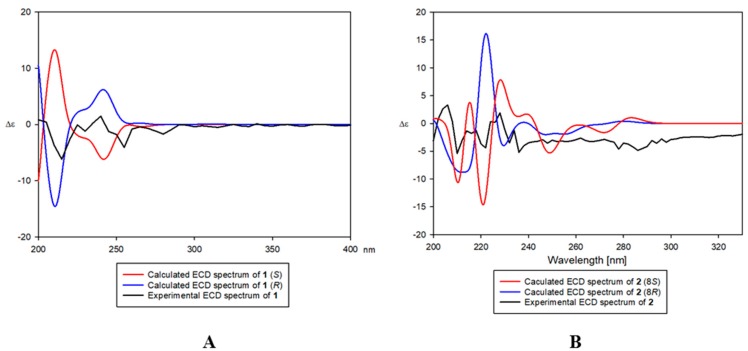
Comparison of experimental and calculated electronic circular dichroism (ECD) spectra of compounds **1** (**A**) and **2** (**B**).

The absolute configuration at C-7′ of compound **1** was established by comparing its experimental ECD spectrum with those calculated spectra of (7′*R*) and (7′*S*) models using the time-dependent density functional theory (TDDFT) method. The experimental ECD spectrum of compound **1** exhibited a positive Cotton effect (CE) at 239 nm (Δε +2.1) and negative CEs at 214 nm (Δε −10.4) and 254 nm (Δε −5.3). The experimental data (Figure 3) was in agreement with the calculated ECD spectrum of the (7′*R*) model, suggesting the absolute configuration of compound **1** as (7′*R*). Thus, the structure of **1** was elucidated as (*R*)-9-(3, 4-dihydroxyphenyl)-6,7-dihydroxy-4,9-dihydronaphtho[2¨C-*c*]furan-1(3*H*)-one, and was named as petasitesin A.

**Figure 4:**
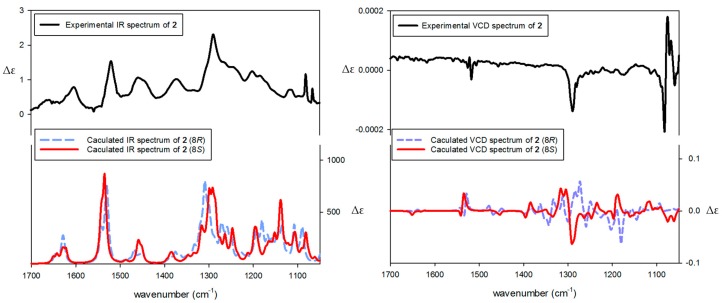
Comparison of experimental and calculated vibrational circular dichroism (VCD) spectra of compound **2** (*c* 0.5 M, DMSO-*d*_6_).

The compound **2** was isolated as a dark brownish powder. Its molecular formula was established as C_18_H_16_O_7_ by HRESIMS (*m/z* 343.0810 [M−H]^−^; calculated for C_18_H_15_O_7_, 343.0818). The ^1^H and ^13^C NMR data of **2** were similar to those of **1** (Table 1), although the NMR solvents were different from each other due to the different solubility of the compounds. Comparison of the ^13^C NMR data and molecular weights from **1** and **2** suggested that the carbons C-8 and C-8′ of **1** with a double bonded linkage (*δ*_C_ 160.9 and 128.2) were replaced by an oxygenated quaternary (*δ*_C_ 78.0) and methine (*δ*_C_ 56.2) carbon atoms. The correlation spectroscopy (COSY) correlation between H-7′ (*δ*_H_ 4.18) and H-8′ (*δ*_H_ 3.24), and the HMBC experiment revealed aryltetralin lactone type lignan (Figure 4). Considering a biogenetic relationship with **1**, the absolute configuration at C-7′ of **2** was suggested to be (*R*)-configuration [15]. The coupling constant of 3.0 Hz between H-7′ and H-8′ suggested the *cis*-geometry of C-7′ and C-8′. It was further confirmed by the NOESY interaction of H-7′ and H-8′ [16]. 

To determine the absolute configuration C-8 of **2,** experimental ECD spectrum of **2** was compared with the calculated spectra of (8*R,*7′*R,*8′*R*) and (8*S,*7′*R,*8′*R*) models using the TDDFT method. The experimental ECD spectrum of **2** showed positive CEs at 203 nm (Δε +4.0) and 229 nm (Δε +1.9), and negative CEs at 210 nm (Δε −5.4) and 222 nm (Δε −4.4). The experimental spectrum (Figure 3) was also in agreement with the calculated ECD spectrum of (8*S,*7′*R,*8′*R*) model. Moreover, the VCD spectrum of **2** was measured additionally to establish the configuration at C-8. The conformity of the experimental IR and VCD spectra and theoretical spectra of **2** suggested the absolute configuration of **2** as (8*S,*7′*R,*8′*R*) (Figure 4). Therefore, the structure of **2** was proposed as (9*R*,3a*S*,9a*R*)-9-(3,4-dihydroxyphenyl)-6,7,3a-trihydroxy-4,9,3a,9a-tetrahydronaphtho[2,3-*c*]furan-1(3*H*)-one, and was named as petasitesin B. 

Compounds **3**–**8** were identified as cimicifugic acid D (**3**) [17], fukinolic acid (**4**) [7], 3,4-dicaffeoylquinic acid (**5**) [18], 3,5-dicaffeoylquinic acid (**6**) [18], 4,5-dicaffeoylquinic acid (**7**) [18], and caffeic acid (**8**) [19] by comparison of their NMR data with those reported.

### 3.2. Anti-inflammatory Effects of the Isolates

**Figure 5:**
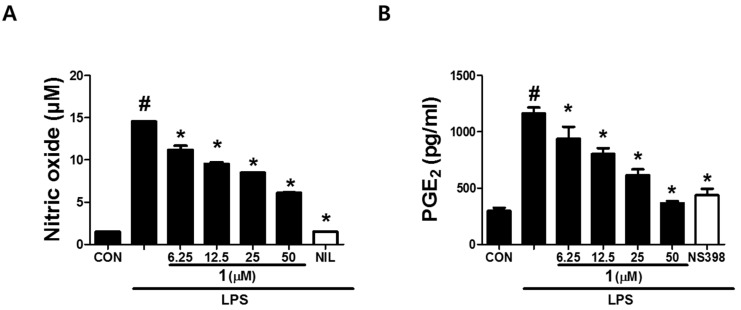
Effects of compound **1** (6.25, 12.5, 25 or 50 *μ*M) on LPS-stimulated NO (**A**) and PGE_2_ (**B**) productions in RAW 264.7 macrophages. #: *p* < 0.05 as compared with the untreated group, *: *p* < 0.05 as compared with the LPS only-treated group.

**Figure 6:**
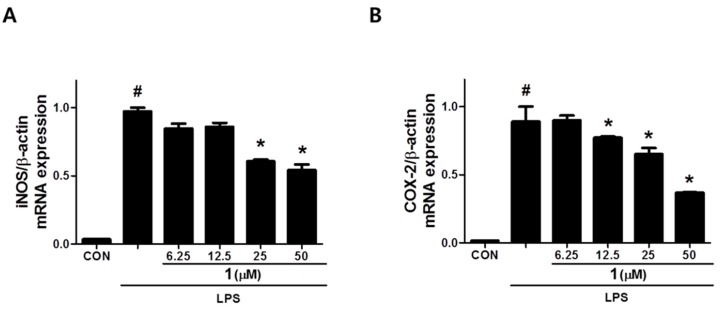
Effect of compound **1** on the expression of iNOS (**A**) and COX-2 (**B**) in RAW 264.7 macrophages. #: *p* < 0.05 as compared with the untreated group, *: *p* < 0.05 as compared with the LPS only-treated group.

As shown in Table 2, cimicifugic acid D (**3**) and the new compound **1** (petasitesin A) exhibited significant inhibitory activities against NO production with IC_50_ values of 12 ± 1.1 and 15 ± 1.4 μM, respectively, without affecting the cell viability (Figure S15). 4,5-Dicaffeoylquinic acid showed mild activity with an observed IC_50_ value of 38.9 ± 0.72 μM. On the other hand, compound **1** showed the most potent inhibitory effect on PGE_2_ production with an IC_50_ value of 17 ± 3.2 μM (Table 2) in a dose-dependent manner (Figure 5). These results suggest that compound **1** might have an anti-inflammatory effect due to inhibition of the production of NO and PGE_2_ which are the key inflammatory mediators of macrophages. It is worth noting that compound **1** significantly suppressed the expression of NO and PGE_2_ synthesis enzymes, inducible nitric oxide synthase (iNOS) and cyclooxygenase-2 (COX-2), respectively (Figure 6), in a concentration-dependent manner. The data indicate that the inhibitory effect of compound **1** on NO and PGE_2_ production in macrophages is related to the regulation of iNOS and COX-2 expression. 

**Figure 7:**
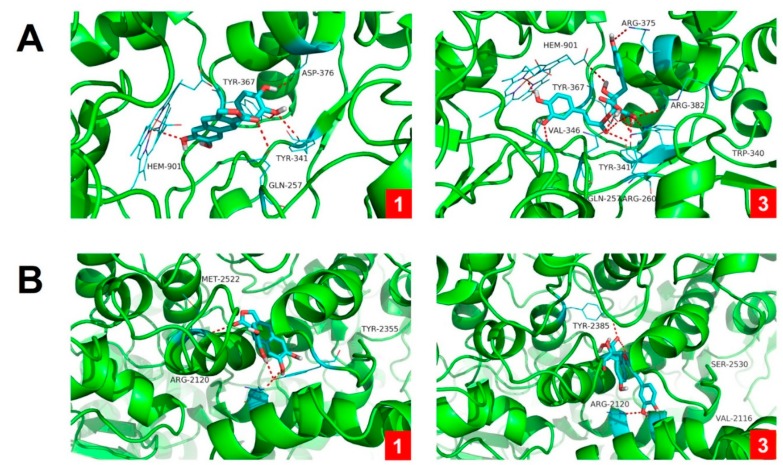
Molecular docking results of compounds **1** and **3** with iNOS (**A**) and COX-2 (**B**) enzymes. Molecular docking simulations obtained at the lowest energy conformation, highlighting potential hydrogen contacts of **1** and **3**, respectively (nitrogen is blue; oxygen is red; carbon is cyan; hydrogen is gray). For clarity, only interacting residues are labeled. Hydrogen bonding interactions are shown by dashes. These figures were created by PyMOL (Schrödinger, LLC, New York, NY, USA: version 1.3).

To better understand the molecular mechanism of inhibitory activities against NO and PGE_2_ production, the most active compounds **1** and **3** were subjected to molecular docking studies. The results showed that **1** and **3** had strong affinities with both NO and PGE_2_ synthesis enzymes, iNOS and COX-2 (Figure 7, Table 3). The binding residues and logarithms of free binding energy are given in Table 3. These results implicated that **1** and **3** may directly interact with the cavity residues of iNOS and COX-2, leading to the activity reduction of free iNOS and COX-2 enzymes. Taken together, these results indicate petasitesin A (**1**), a novel lignan isolated from butterbur leaves extract, exhibits anti-inflammatory properties by suppressing NO and PGE_2_ production via inhibiting the expression of iNOS and COX-2 and binding to the free iNOS and COX-2 enzymes.

## 4. Discussion

In the present study, we isolated two new aryltetralin lactone lignans, petasitesin A and B (**1** and **2**) from the leaves of *P. japonicus*. To the best of our knowledge, this is the first report on the isolation of the aryltetralin lactone type lignans from the leaves of *P. japonicas*. Although cimicifugic acid D (**3**) has been isolated from *Cimicifuga* spp. including black cohosh (*Cimicifuga racemosa*) and possesses vasoactive effect and hyaluronidase inhibitory activity [20,21], this is the first finding that it presents in *P. japonicus* and inhibits pro-inflammatory mediators, NO and PGE_2_. 

A new lignan petasitesin A (**1**) showed a potent inhibitory effect on the production of both NO and PGE_2_ in LPS-stimulated macrophages (IC_50_ values < 20 μM). Our molecular docking studies reveal that petasitesin A (**1**) can interact with the cavity residues of both iNOS and COX-2. Interestingly, petasitesin A (**1**) also inhibited the mRNA expression of iNOS and COX-2 induced by LPS in macrophages. However, the molecular mechanism of action underlying the gene expression regulation by petasitesin A remains to be investigated. Considering that LPS binds to toll-like receptor 4 (TLR4), the TLR4-mediated NF-κB pathway is likely associated with the inhibition of iNOS and COX-2 expression by petasitesin A. In fact, NF-κB is a key transcriptional factor to regulate the iNOS and COX-2 gene in macrophages under the inflammatory condition. In this regard, the effect of petasitesin A on the NF-κB pathway can be further elucidated.

## 5. Conclusions

New lignans (compounds **1** and **2**) and six known compounds were isolated and identified from the leaves of *P. japonicus*. Petasitesin A (**1**) and cimicifugic acid D (**3**) inhibit production of inflammatory mediators NO and PGE_2_. PetasitesinA (**1**) inhibits iNOS and COX-2 expression, and petasitesin A (**1**) and cimicifugic acid D (**3**) have strong affinities with both iNOS and COX-2 enzymes in molecular docking studies. Thus, petasitesin A (**1**) and cimicifugic acid D (**3**) are worthy of further pharmacological evaluation for their potential as anti-inflammatory drugs.

## Figures and Tables

**Table 1:** ^1^H (500 MHz) and ^13^C NMR (125 MHz) data of compounds **1** and **2**.

Position	1 (in Acetone-*d*_6_)		2 (in CD_3_OD)	
	*δ*_C_	*δ*_H_ Multi (*J* in Hz)	*δ*_C_	*δ*_H_ Multi (*J* in Hz)
1	129.8		127.0	
2	136.9		130.1	
3	116.9	6.60 s	117.1	6.55 s
4	145.1		145.5	
5	145.3		145.7	
6	115.7	6.72 s	117.0	6.64 s
7	29.0	3.86 d (23.0)	39.6	2.83 d (14.5)
		3.62 overlapped		2.67 d (14.5)
8	160.9		78.0	
9	72.4	4.97 d (17.0)	80.1	4.17 d (10.0)
		4.89 d (17.0)		3.93 d (10.0)
1′	123.0		135.5	
2′	116.2	6.59 d (2.0)	116.0	6.58 d (2.0)
3′	145.9		146.3	
4′	144.6		144.8	
5′	116.1	6.63 d (8.0)	116.2	6.68 d (8.0)
6′	120.3	6.45 dd (8.0, 2.0)	120.2	6.52 dd (8.0, 2.0)
7′	42.3	4.53 s	47.4	4.18 d (3.0)
8′	128.2		56.2	3.24 d (3.0)
9′	173.9		181.0	

**Table 2:** Inhibitory effects of the compounds from *P. japonicus* on NO and PGE_2_ production in lipopolysaccharide (LPS)-induced RAW 264.7 cells.

Compound	IC_50_ (μM)*^a^*	
	NO	PGE_2_
**1**	15 ± 1.4	17 ± 3.2
**2**	>50	>50
Cimicifugic acid D (**3**)	12 ± 1.1	43 ± 7.9
4,5-Dicaffeoylquinic acid	38.9 ± 0.72	>50
Caffeic acid	>50	45.7 ± 0.87

*^a^* The values represent the means of the results from three independent experiments with similar patterns. l-*N*^6^-(1-Iminoethyl)lysine (l-NIL) and *N*-[2-(cyclohexyloxy)-4-nitrophenyl]methanesulfonamide (NS-398) were used as a positive control substance for NO [half maximal inhibitory concentration IC_50_) value = 1.62 ± 0.08 μM] and prostaglandin E_2_ (PGE_2_) productions (IC_50_ value = 3.3 ± 0.15 μM), respectively. Three known compounds, fukinolic acid, 3,4-dicaffeoylquinic acid, and 3,5-dicaffeoylquinic acid were inactive (IC_50_ value > 50 μM) in this assay system.

**Table 3:** Logarithms of free binding energies (FBE, kcal/ mol) of NO inhibitors to the active cavities of iNOS (PDB Code: 3E6T) and COX-2 (PDB code: 1PXX) and targeting residues of the binding site located on the mobile flap.

Compound	−Log (FBE)		Targeting Residues	
	iNOS	COX-2	iNOS	COX-2
**1**	−8.8	−7.5	ASP-376,TYR-367, TYR-341, GLN-257, HEM-901	ARG-2120, TYR-3355, MET-2522
**3**	−10.0	−8.3	ARG-260, ARG-375, ARG-382 TYR-341, TYR-367, TRP-340, GLN-257, HEM-901, VAL-346	ARG-2120, TYR-2385, VAL-2116, SER-2530

## Supplementary Materials

The following are available online at https://www.mdpi.com/2218-273X/9/12/806/s1, Figure S1: HR-ESI-MS spectrum of compound **1**, Figure S2: The ^1^H-NMR (500 MHz, CD_3_COCD_3_) spectrum of compound **1**, Figure S3: The ^13^C-NMR (125 MHz, CD_3_COCD_3_) spectrum of compound **1**, Figure S4: The HSQC spectrum of compound **1** in CD_3_COCD_3_, Figure S5: The COSY spectrum of compound **1** in CD_3_COCD_3_, Figure S6: The HMBC spectrum of compound **1** in CD_3_COCD_3_, Figure S7: The NOESY spectrum of compound **1** in CD_3_COCD_3_, Figure S8: HR-ESI-MS spectrum of compound **2**, Figure S9: The ^1^H-NMR (500 MHz, CD_3_OD) spectrum of compound **2**, Figure S10: The ^13^C-NMR (125 MHz, CD_3_OD) spectrum of compound **2**, Figure S11: The HSQC spectrum of compound **2** in CD_3_OD, Figure S12: The COSY spectrum of compound **2** in CD_3_OD, Figure S13: The HMBC spectrum of compound **2** in CD_3_OD, Figure S14: The NOESY spectrum of compound **2** in CD_3_OD, Figure S15: Cell viability of the isolates from *P. japonicus*.
