# Supplementary material for: Chemical Constituents of the Leaves of Butterbur (Petasites japonicus) and Their Anti-Inflammatory Effects
Source: Biomolecules. 2019 Nov 29;9(12):806. doi: 10.3390/biom9120806 (PMC6995602; doi:10.3390/biom9120806)
Supplement: Supplementary file 1 [file biomolecules-09-00806-s001.pdf]

Supplementary Materials

Chemical Constituents of the Leaves of Butterbur (*Petasites japonicus*) and their Anti-Inflammatory Effects

Content

General Experimental Procedures
Figure S1. HR-ESI-MS spectrum of compound **1**
Figure S2. The ^{1}H-NMR (500 MHz, CD_3COCD_3) spectrum of compound **1**
Figure S3. The ^{13}C-NMR (125 MHz, CD_3COCD_3) spectrum of compound **1**
Figure S4. The HSQC spectrum of compound **1** in CD_3COCD_3
Figure S5. The COSY spectrum of compound **1** in CD_3COCD_3
Figure S6. The HMBC spectrum of compound **1** in CD_3COCD_3
Figure S7. The NOESY spectrum of compound **1** in CD_3COCD_3
Figure S8. HR-ESI-MS spectrum of compound **2**
Figure S9. The ^{1}H-NMR (500 MHz, CD_3OD) spectrum of compound **2**
Figure S10. The ^{13}C-NMR (125 MHz, CD_3OD) spectrum of compound **2**
Figure S11. The HSQC spectrum of compound **2** in CD_3OD
Figure S12. The COSY spectrum of compound **2** in CD_3OD
Figure S13. The HMBC spectrum of compound **2** in CD_3OD
Figure S14. The NOESY spectrum of compound **2** in CD_3OD
Figure S15. Cell viability of the isolates from *P. japonicus*.

General Experimental Procedures

Optical rotations and UV spectra were obtained on a JASCO P-2000 polarimeter (JASCO, Tokyo, Japan) and Optizen pop instrument (Mecasys, Daejeon, Korea), respectively. ECD and VCD spectra were measured with a J-2200 circular dichroism spectrophotometer (JASCO) and a ChiralIR-2X TM FT-VCD spectrometer (BioTools, Jupiter, FL, USA), respectively. ECD and VCD spectra were calculated by Spartan'14 (Wavefunction, Inc., Irvine, CA, USA; 2014) and Gaussian 09 (Revision E.01; Gaussian, Inc., Wallingford, CT, USA; 2009). IR spectra were obtained on an Agilent Cary 630 FTIR spectrometer (Agilent Technologies, Santa Clara, CA, USA). NMR and HRESIMS spectra were obtained using a JEOL 500 MHz (JEOL, Tokyo, Japan) and a Q-TOF micro mass spectrometer (Waters, Milford, MA, USA), respectively. TLC analysis was performed on Silica gel 60 F_{254} (Merck, Kenilworth, NJ, USA) and RP-18 F_{254S} (Merck) plates. Sephadex LH-20 (Amersham Pharmacia Biotech, Buckinghamshire, United Kingdom), Diaion HP-20 (Mitsubishi, Tokyo, Japan), and LiChroprep RP-18 (Merck, 40–63 μm) were used for column chromatography. MPLC and HPLC were performed using the flash purification system (Combi Flash Rf, Teledyne Isco, Lincoln, NE, USA) with Pre-packed cartridges, Redi Sep-C18 (13 g, 26 g, 43 g, 130 g, Teledyne Isco) and the Gilson purification system with a YMC Pack ODS-A column (250 × 20.0 mm i.d., 5.0 μm, YMC Co., Tokyo, Japan) and a Luna 10 μm C18(2) 100A column (250 × 21.2 mm i.d., 10.0 μm, Phenomenex, Torrance, CA, USA), respectively. All solvents used for the chromatographic separations were distilled before use.

Single Mass Analysis
Tolerance = 5.0 PPM / DBE: min = -1.5, max = 50.0
Selected filters: None
Monoisotopic Mass, Even Electron Ions
61 formula(e) evaluated with 1 results within limits (up to 50 best isotopic matches for each mass)

Mass	Calc. Mass	mDa	PPM	DBE	Formula	i-FIT	C	H	O
325.0714	325.0712	0.2	0.6	12.5	C18 H13 O6	29.2	18	13	6

Molecular formula : $C_{18}H_{14}O_6$

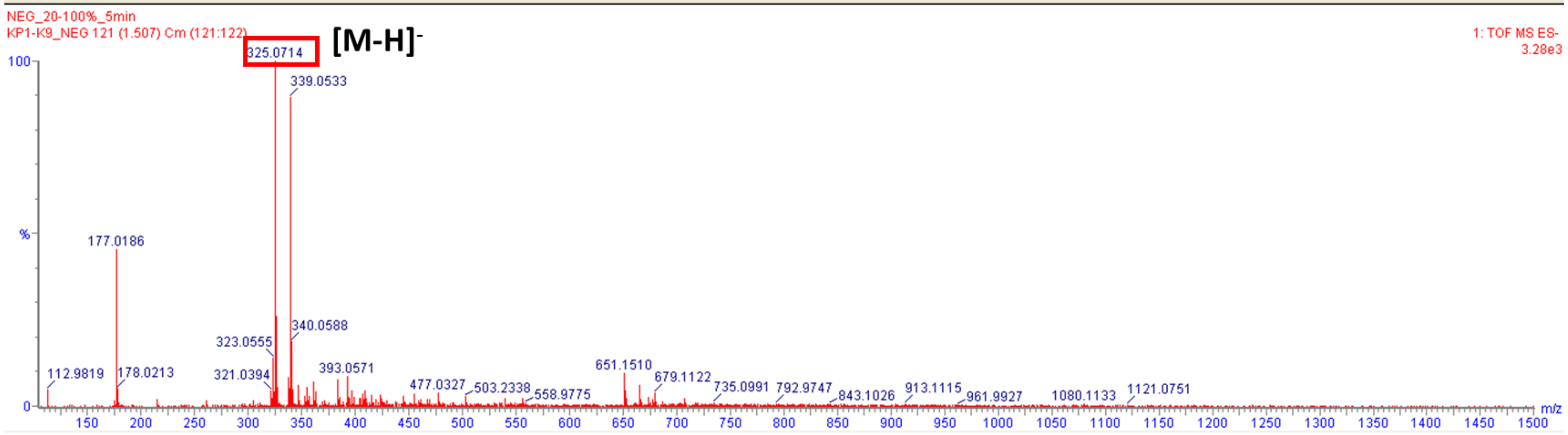

Figure S1. HR-ESI-MS spectrum of compound **1.**

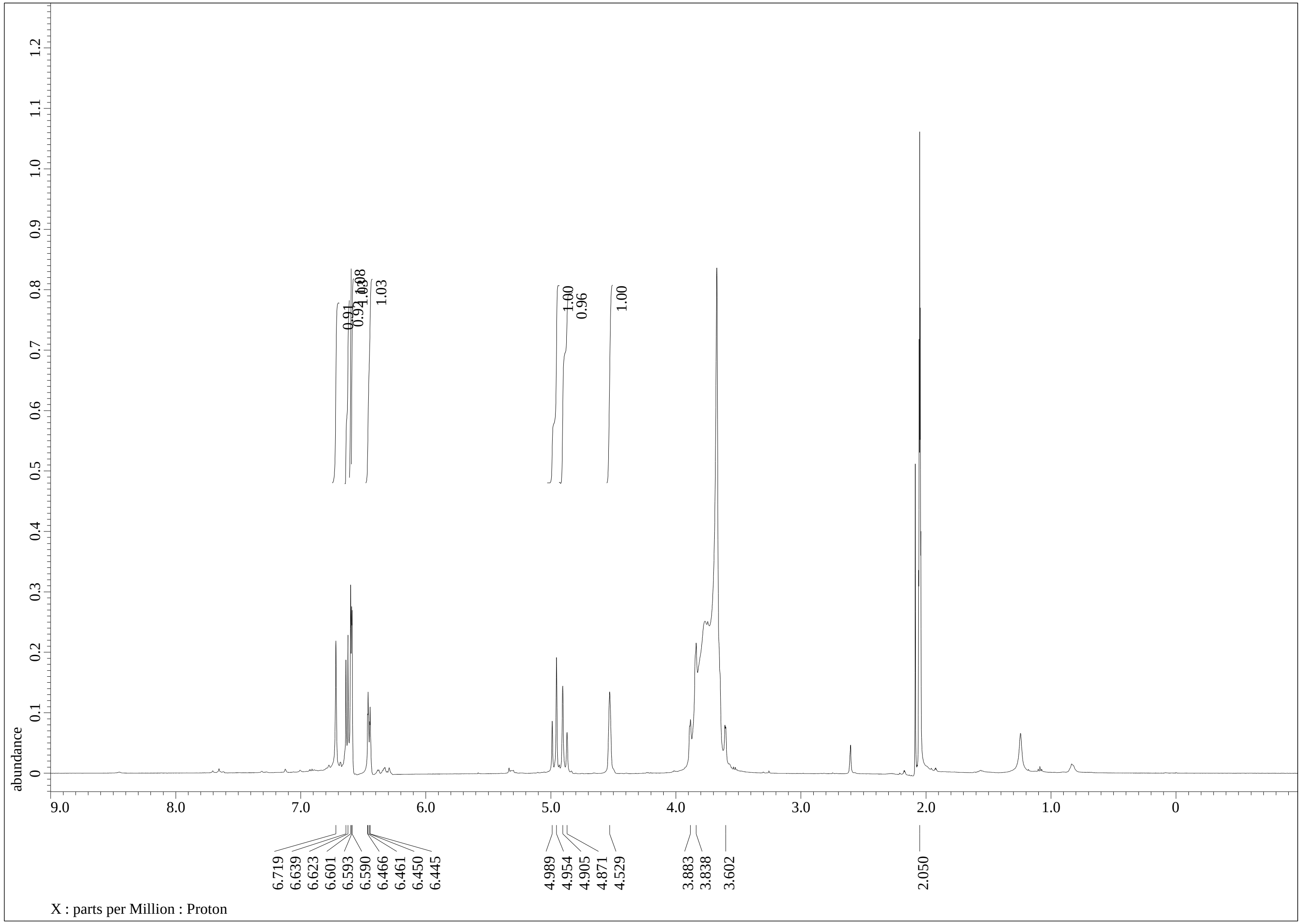

Figure S2. The ^{1}H-NMR (500 MHz, CD_3OCD_3) spectrum of compound **1.**

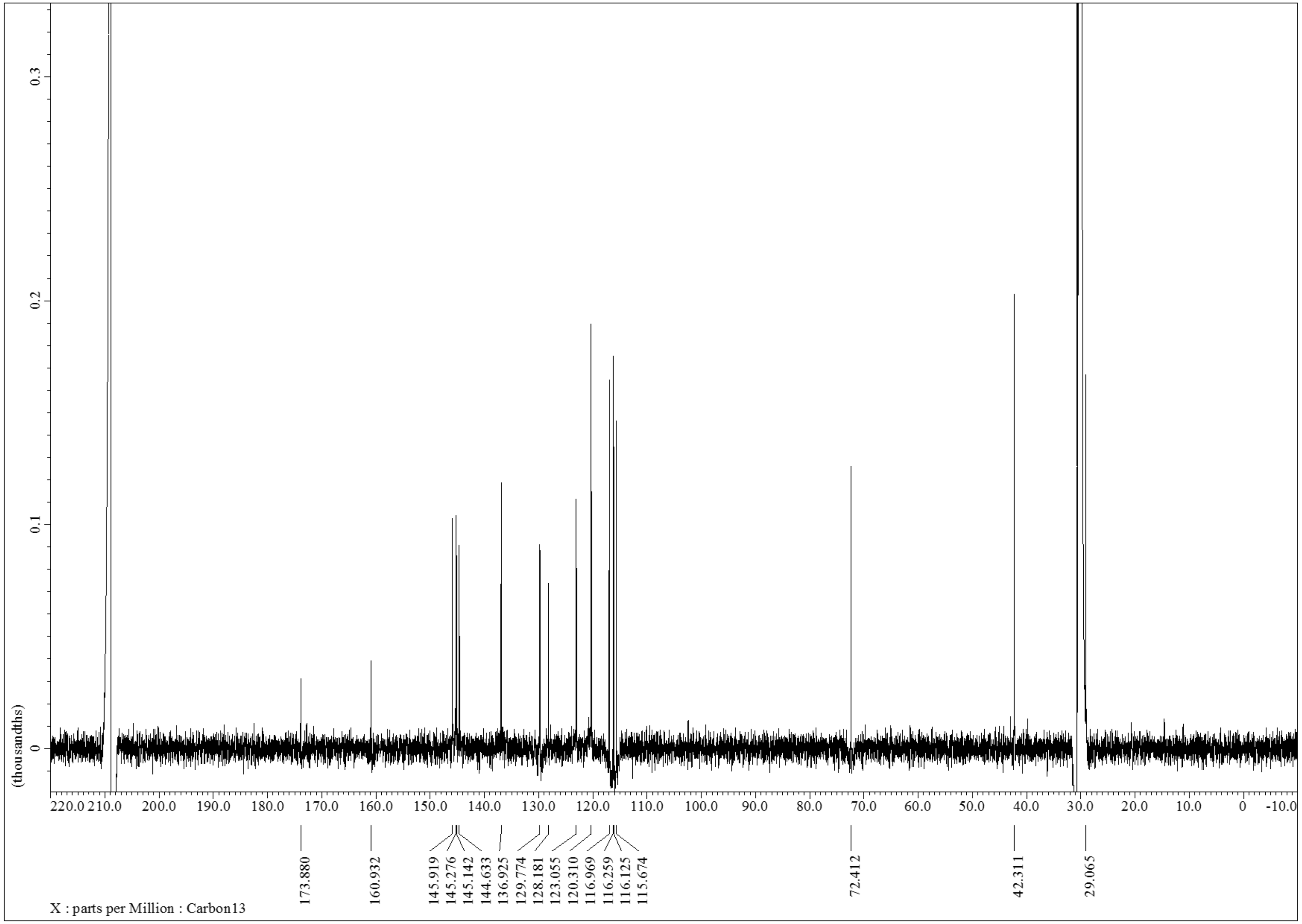

Figure S3. The ^{13}C-NMR (125 MHz, CD_3OCD_3) spectrum of compound 1.

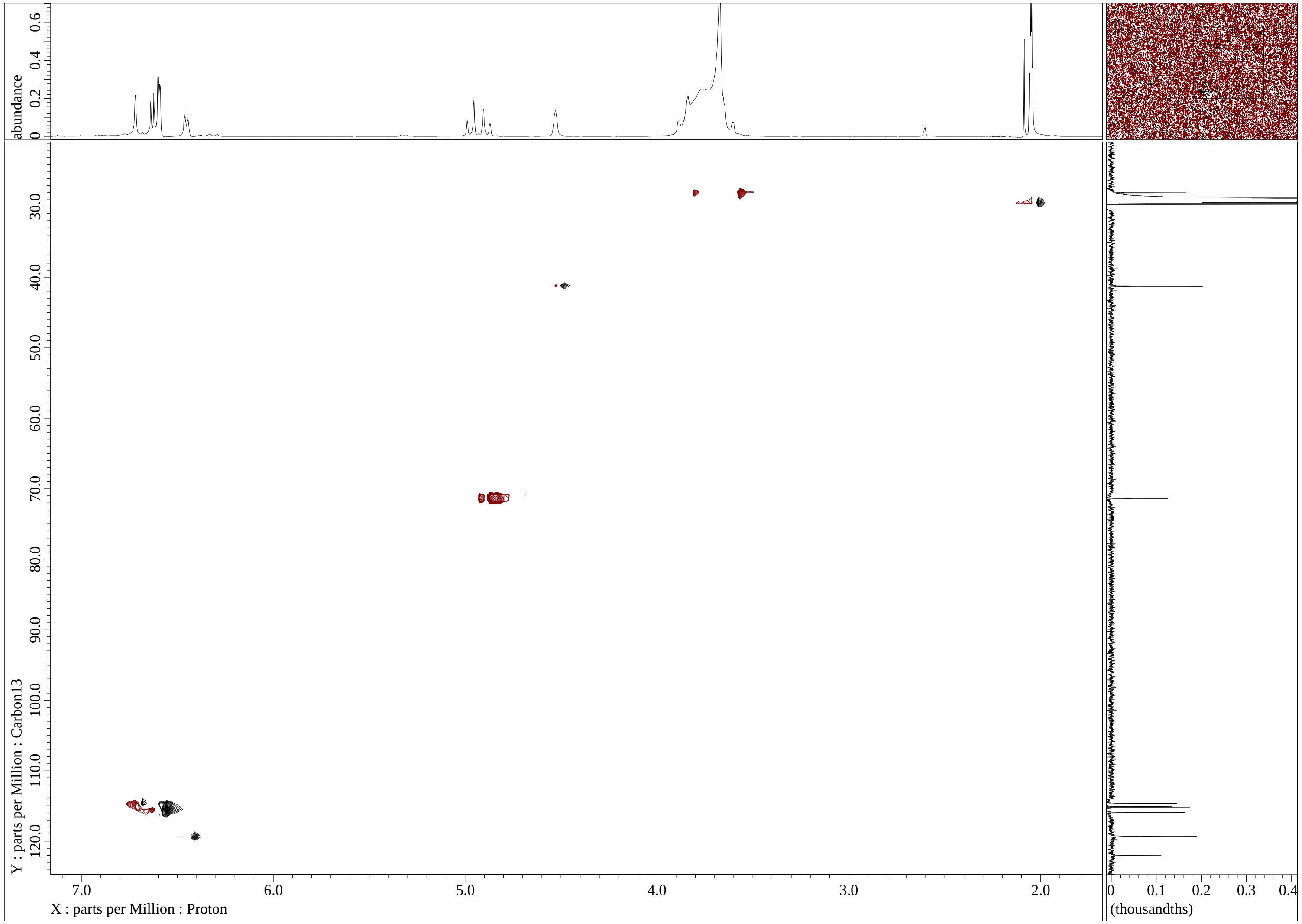

Figure S4. The HSQC spectrum of compound **1** in CD_3OCD_3.

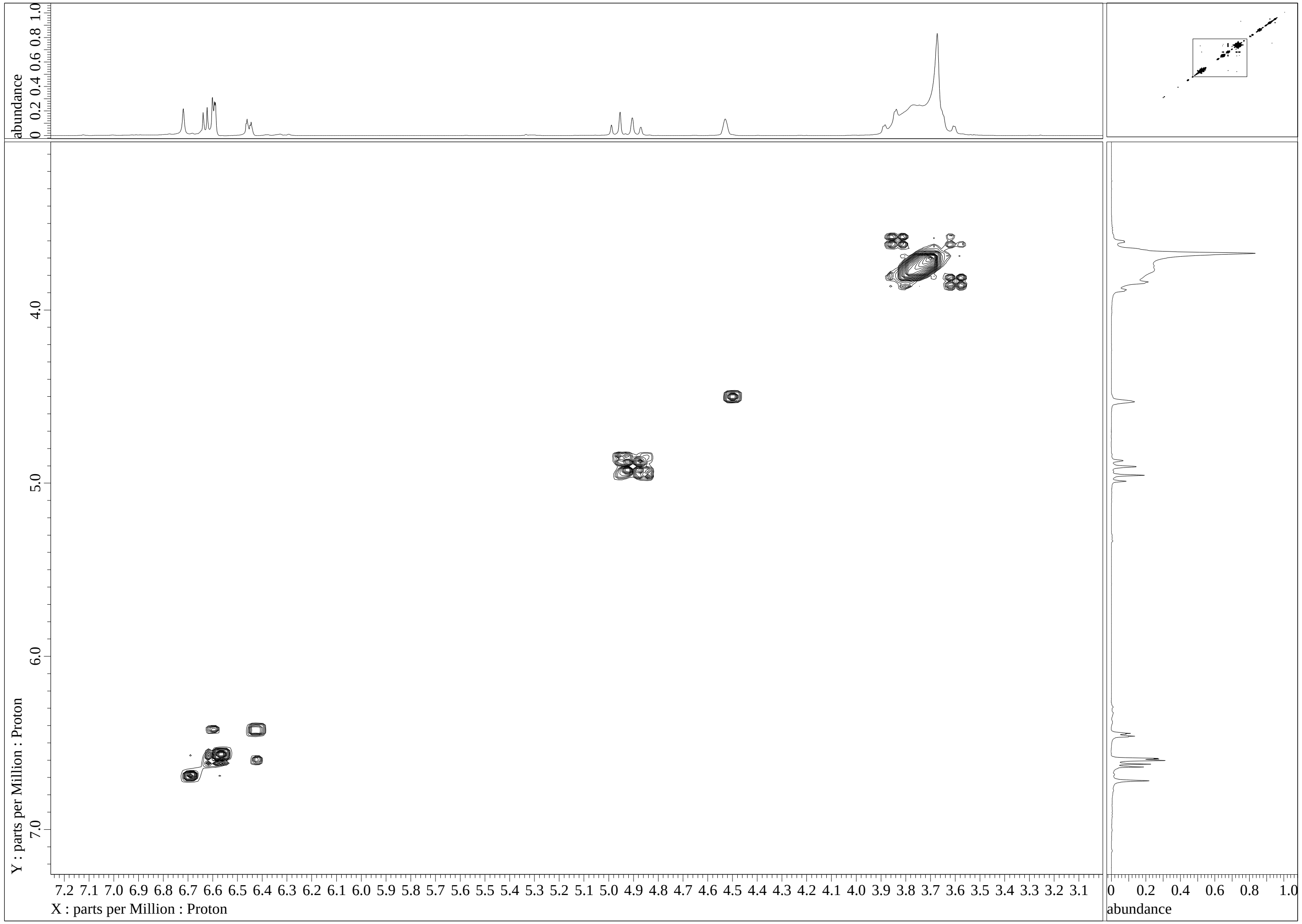

Figure S5. The COSY spectrum of compound **1** in CD_3OCD_3.

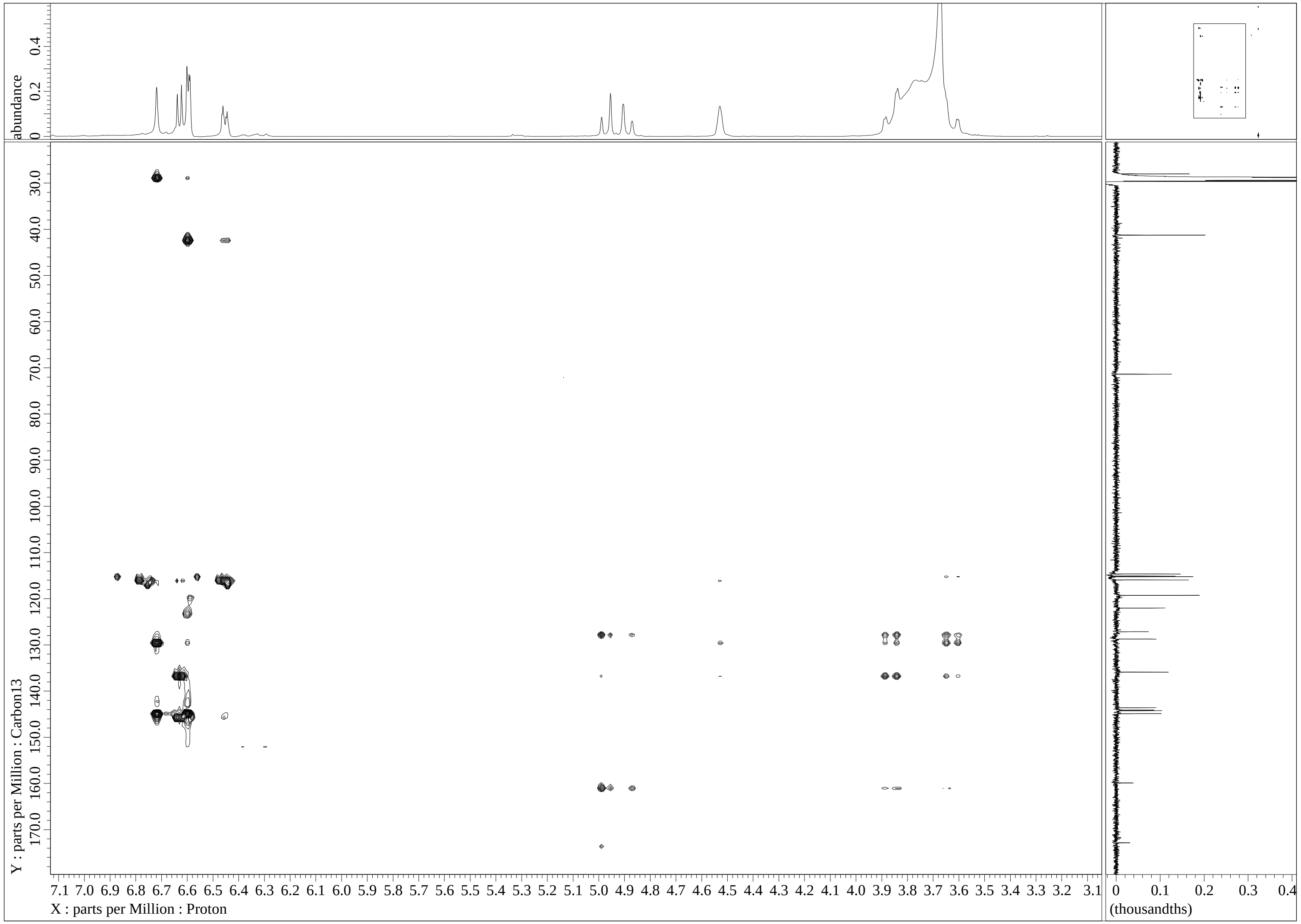

Figure S6. The HMBC spectrum of compound **1** in CD_3OCD_3.

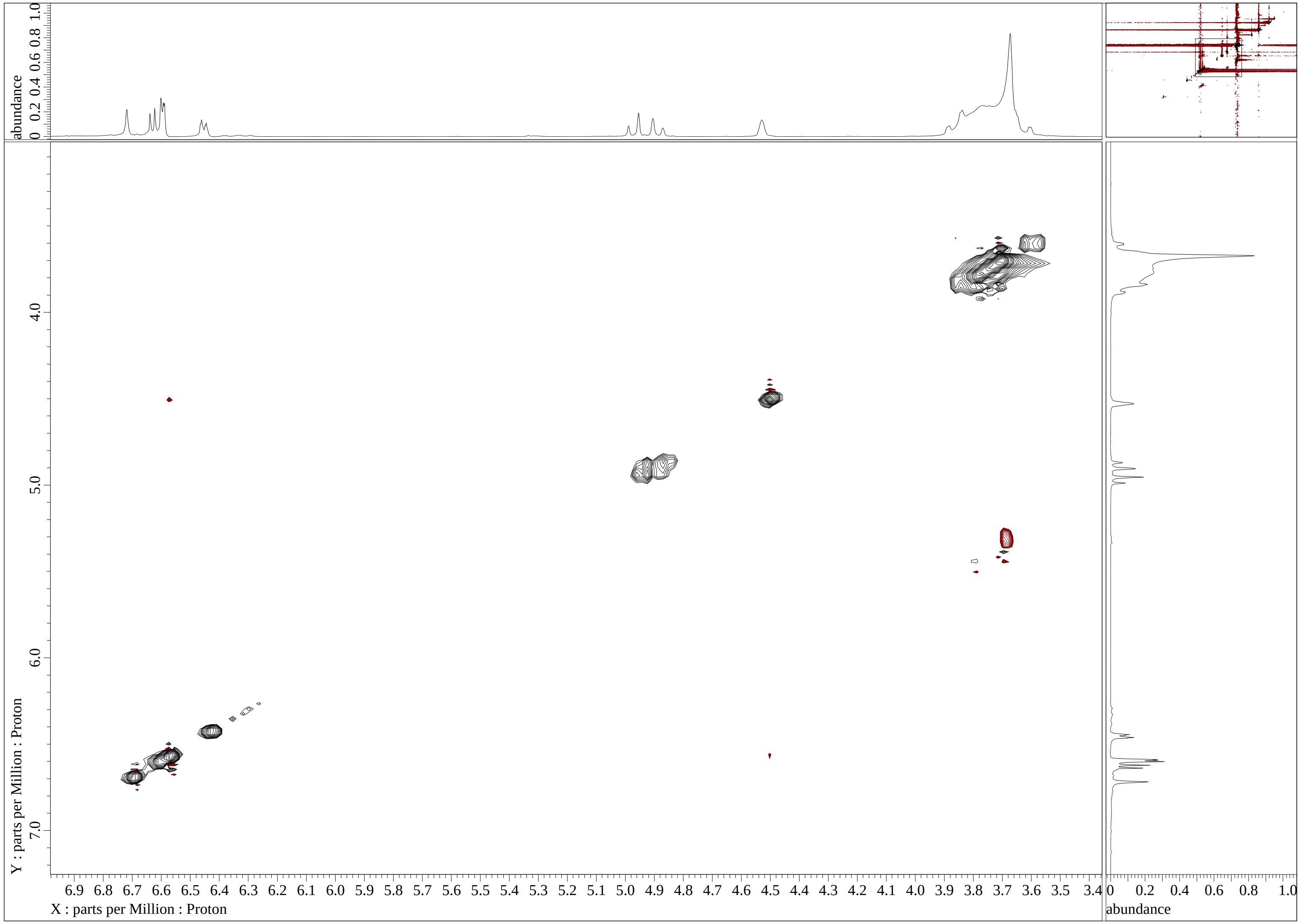

Figure S7. The NOESY spectrum of compound **1** in CD_3OCD_3.

Single Mass Analysis
Tolerance = 5.0 PPM / DBE: min = -1.5, max = 50.0
Selected filters: None
Monoisotopic Mass, Even Electron Ions
70 formula(e) evaluated with 1 results within limits (up to 50 best isotopic matches for each mass)

Mass	Calc. Mass	mDa	PPM	DBE	Formula	i-FIT	C	H	O
343.0810	343.0818	-0.8	-2.3	11.5	C18 H15 O7	3.6	18	15	7

Molecular formula : $C_{18}H_{16}O_7$

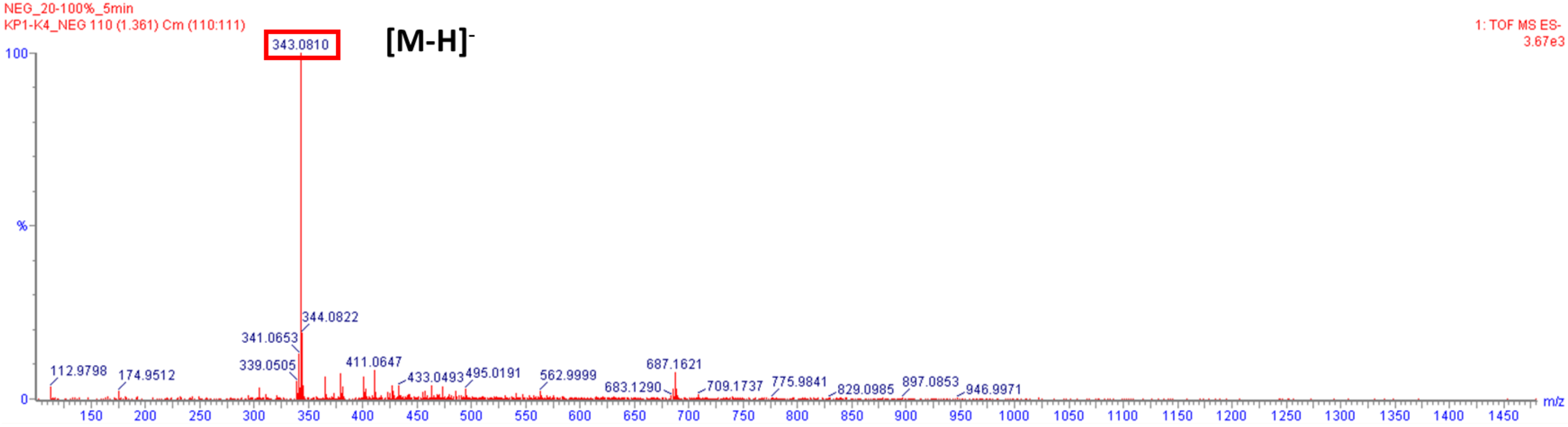

Figure S8. HR-ESI-MS spectrum of compound **2.**

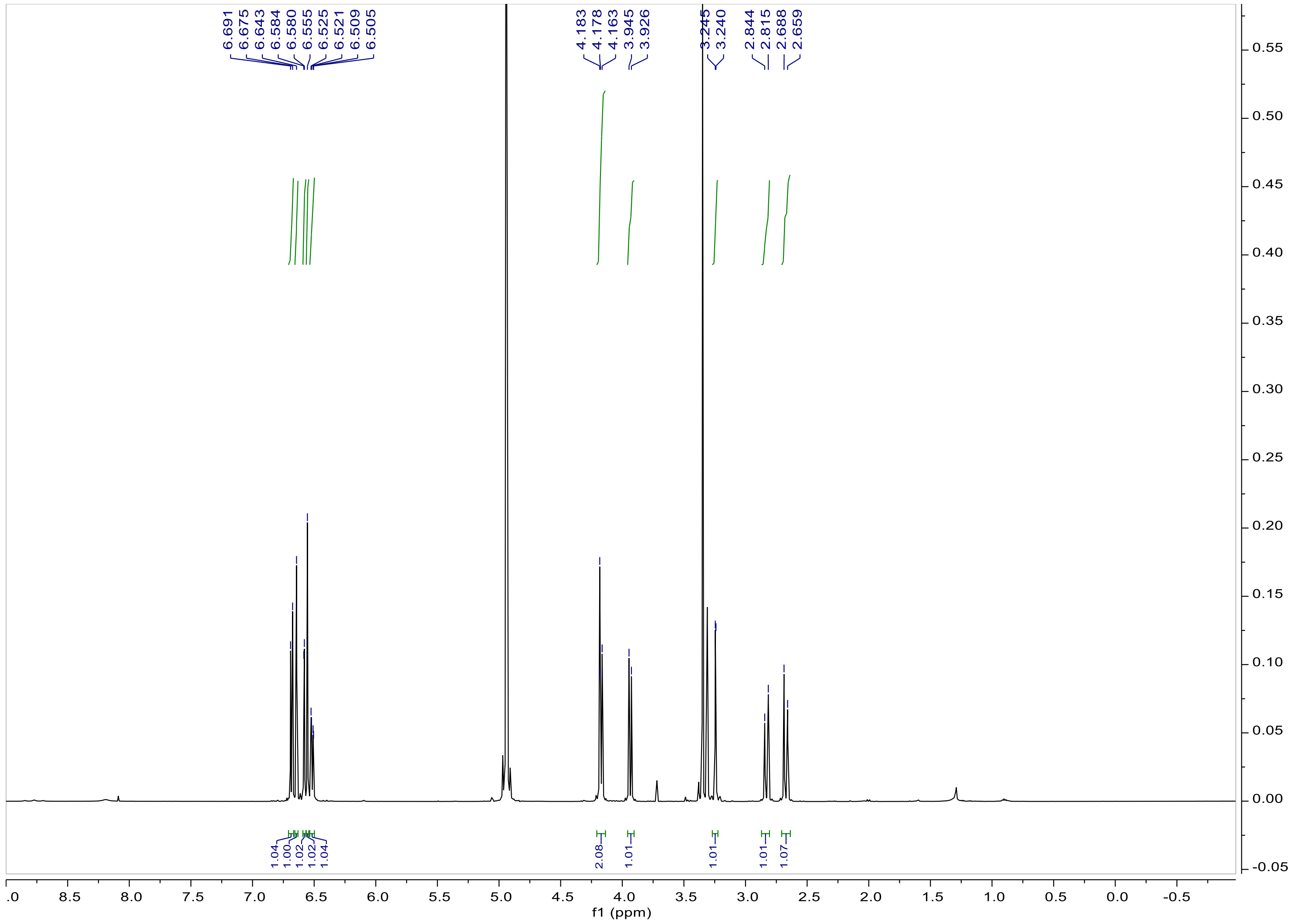

Figure S9. The ¹H-NMR (500 MHz, CD_3OD) spectrum of compound **2.**

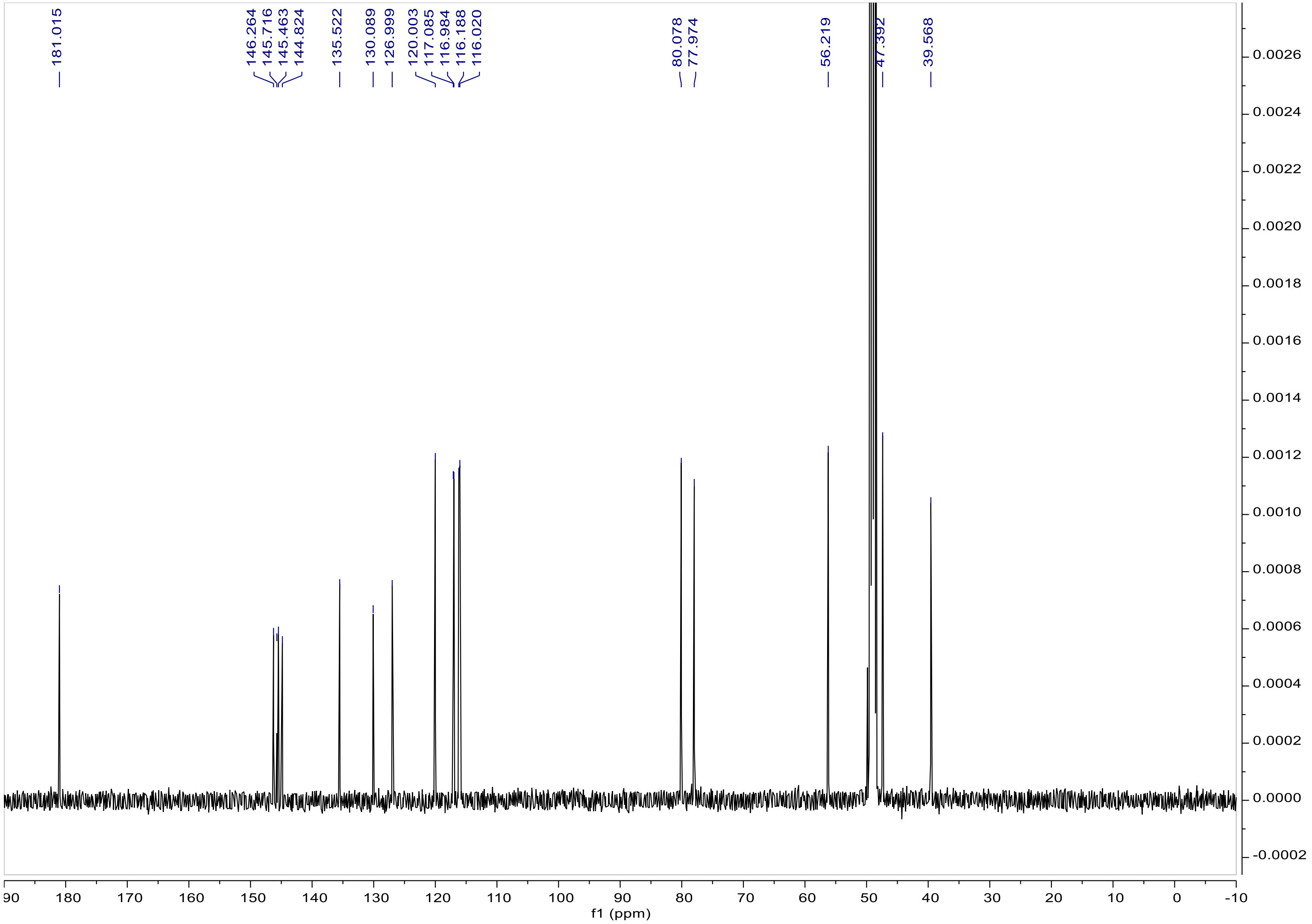

Figure S10. The ^{13}C-NMR (125 MHz, CD_3OD) spectrum of compound **2.**

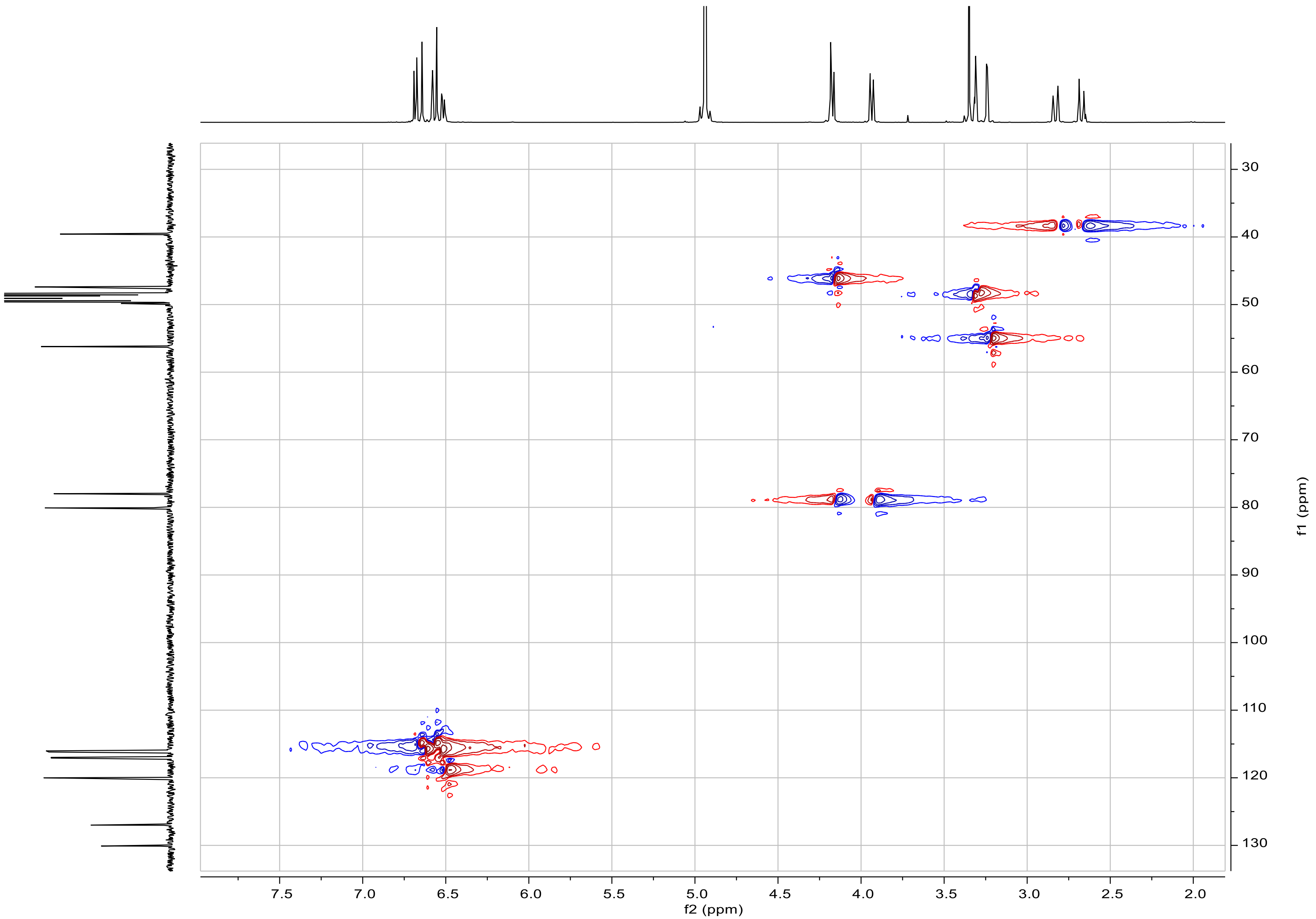

Figure S11. The HSQC spectrum of compound **2** in CD_3OD.

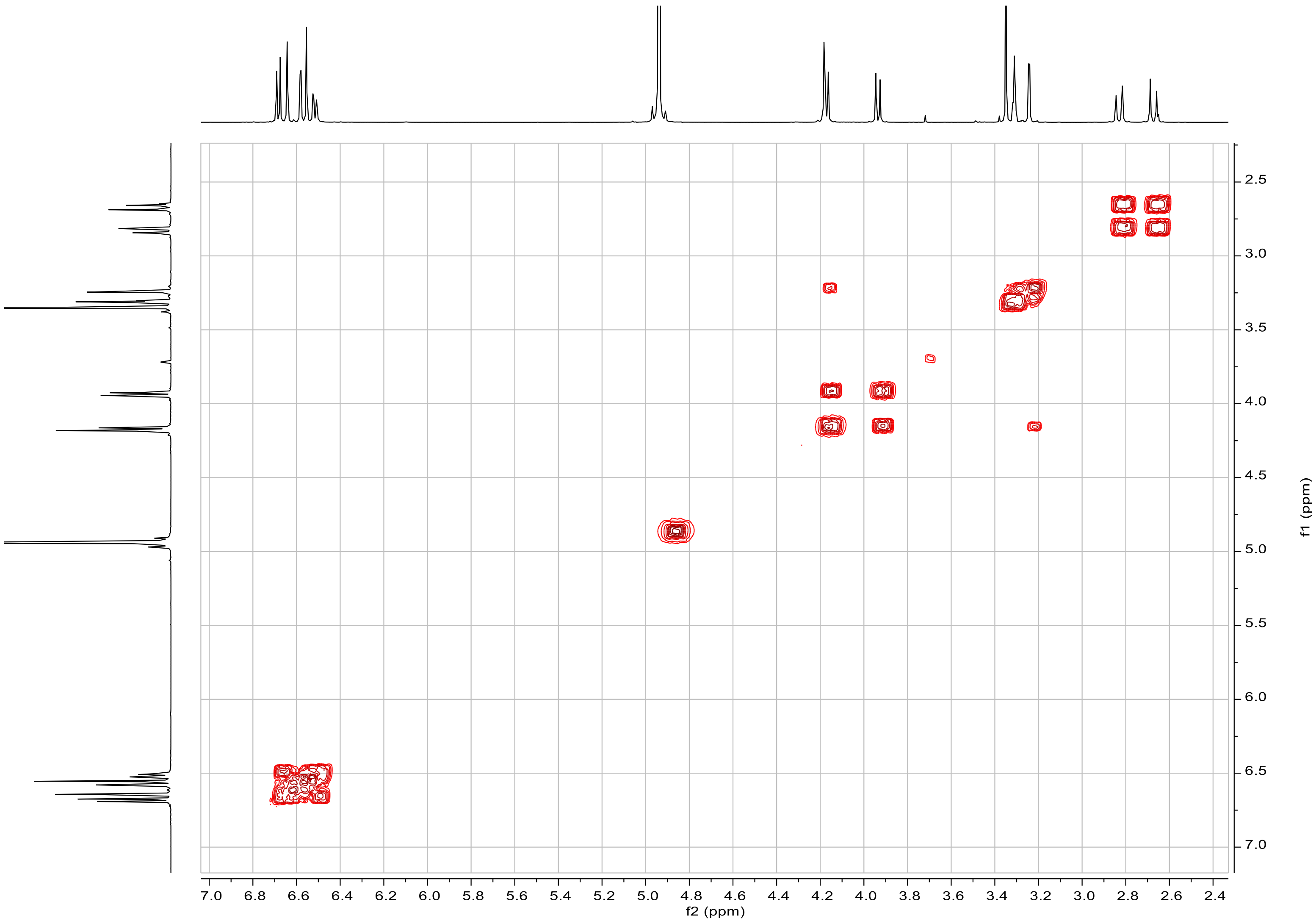

Figure S12. The COSY spectrum of compound **2** in CD_3OD.

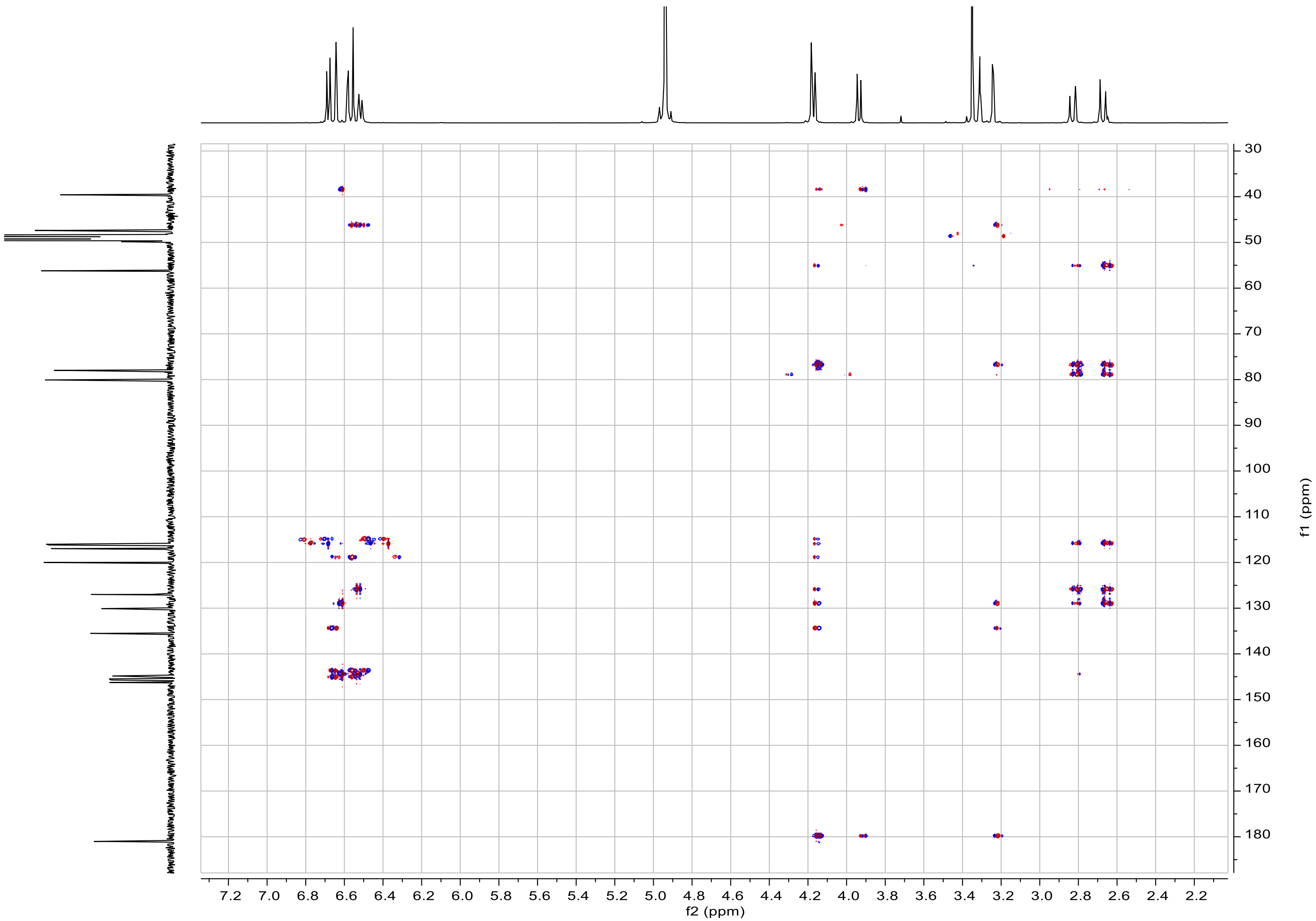

Figure S13. The HMBC spectrum of compound **2** in CD_3OD.

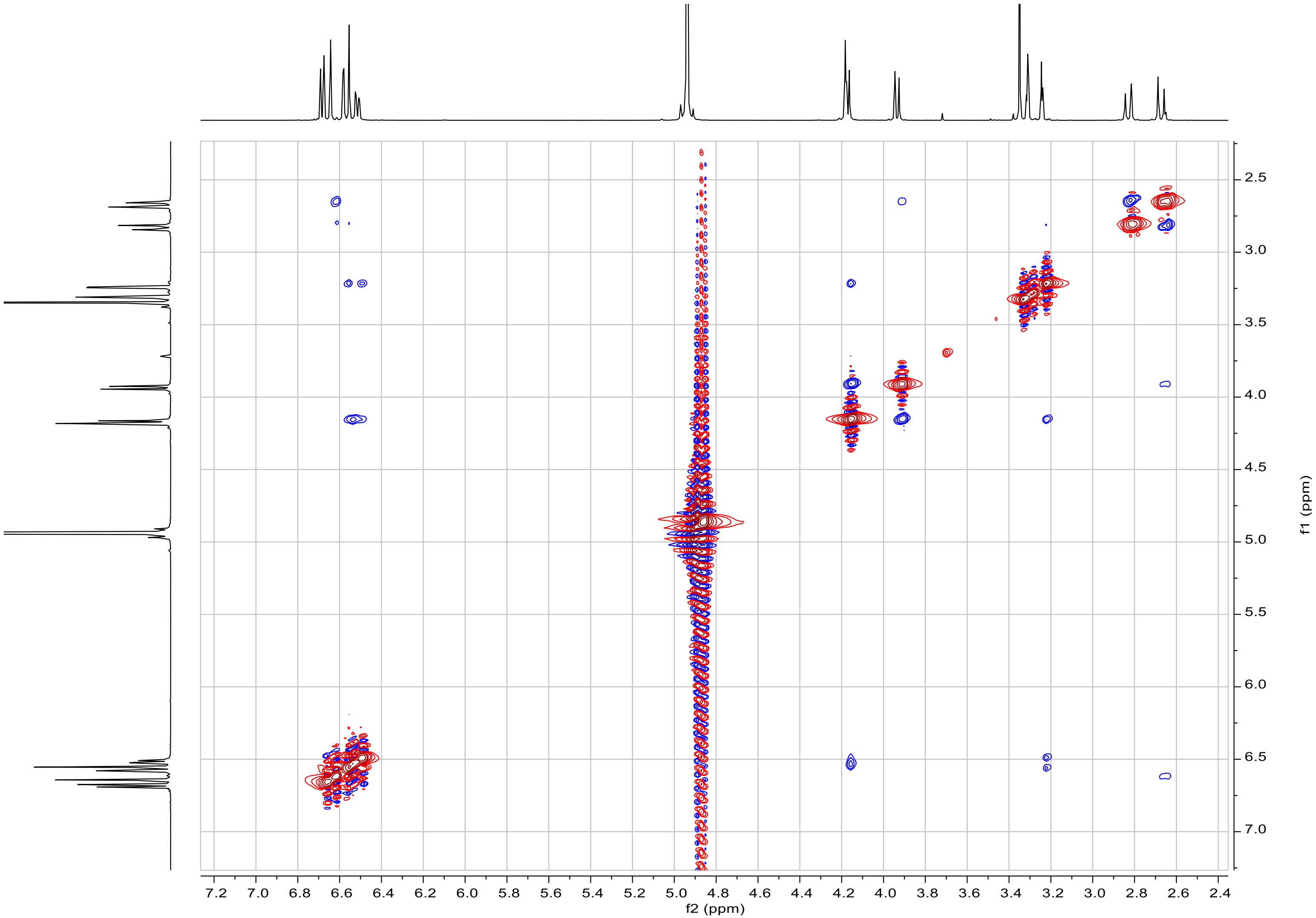

Figure S14. The NOESY spectrum of compound **2** in CD_3OD.

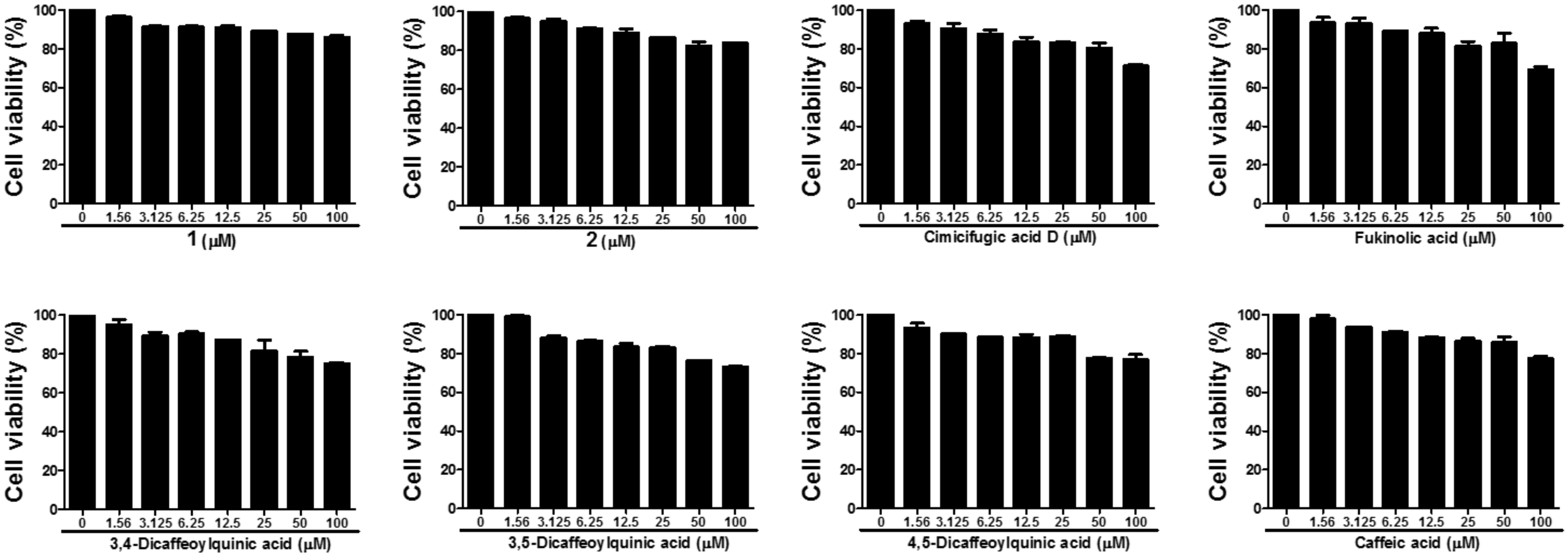

Figure 15. Cell viability of the isolates from *P. japonicus*.
